# Dissection of a novel major stable QTL on chromosome 7D for grain hardness and its breeding value estimation in bread wheat

**DOI:** 10.3389/fpls.2024.1356687

**Published:** 2024-02-01

**Authors:** Xiaofeng Liu, Zhibin Xu, Bo Feng, Qiang Zhou, Shaodan Guo, Simin Liao, Yuhao Ou, Xiaoli Fan, Tao Wang

**Affiliations:** ^1^ Chengdu Institute of Biology, Chinese Academy of Sciences, Chengdu, China; ^2^ Insitute of Plant Protection, Sichuan Academy of Agricultural Science, Chengdu, China; ^3^ University of Chinese Academy of Sciences, Beijing, China; ^4^ Innovative Academy for Seed Design, Chinese Academy of Sciences, Beijing, China

**Keywords:** grain hardness, quality-related traits, QTL mapping, BSE-Seq, Puroindoline

## Abstract

Grain hardness (Gh) is important for wheat processing and end-product quality. *Puroindolines* polymorphism explains over 60% of Gh variation and the novel genetic factors remain to be exploited. In this study, a total of 153 quantitative trait loci (QTLs), clustered into 12 genomic intervals (C1-C12), for 13 quality-related traits were identified using a recombinant inbred line population derived from the cross of Zhongkemai138 (ZKM138) and Chuanmai44 (CM44). Among them, C7 (harboring eight QTLs for different quality-related traits) and C8 (mainly harboring *QGh.cib-5D.1* for Gh) were attributed to the famous genes, *Rht-D1* and *Pina*, respectively, indicating that the correlation of involved traits was supported by the pleotropic or linked genes. Notably, a novel major stable QTL for Gh was detected in the C12, *QGh.cib-7D*, with ZKM138-derived allele increasing grain hardness, which was simultaneously mapped by the BSE-Seq method. The geographic pattern and transmissibility of this locus revealed that the increasing-Gh allele is highly frequently present in 85.79% of 373 worldwide wheat varieties and presented 99.31% transmissibility in 144 ZKM138-derivatives, indicating the non-negative effect on yield performance and that its indirect passive selection has happened during the actual breeding process. Thus, the contribution of this new Gh-related locus was highlighted in consideration of improving the efficiency and accuracy of the soft/hard material selection in the molecular marker-assisted process. Further, *TraesCS7D02G099400*, *TraesCS7D02G098000*, and *TraesCS7D02G099500* were initially deduced to be the most potential candidate genes of *QGh.cib-7D*. Collectively, this study provided valuable information of elucidating the genetic architecture of Gh for wheat quality improvement.

## Introduction

Wheat (*Triticum aestivum* L.), one of the most widely planted food crops, provides approximately 20% of the dietary calories in food products consumed worldwide. Grain hardness (Gh) is a key trait contributing to milling quality and the end-use qualities in wheat, decided by the degrees of interaction between starch granules and the protein matrix within the endosperm (Pasha et al., 2010; Miki et al., 2020). According to Gh, wheat can be classified into hard and soft types. Hard wheat exhibits high crushing resistance due to strong adhesion between starch granules and the protein matrix (Pasha et al., 2010). Its flour had more damaged starch and higher water absorption than soft wheat flour (Oury et al., 2015), thus usually suited for making bread and noodles whereas soft wheat is better for cookies, cakes, and pastries (Guttieri et al., 2001). Besides the common pasta quality characteristics are greatly determined by grain hardness, in China, the Gh is also a major determinant for Baijiu (the famous Chinese liquor) flavor and quality, considering that wheat is the major raw material for producing the Jiuqu (Baijiu starter) and thus severely affect the final flavor and commercial value of Baijiu. Especially the soft wheat has higher saccharigying efficiency for simultaneously facilitating asccharification and fermentation, thus it is usually thought to be more suited for starter production of Chinese Baijiu (Wang et al., 2018). Therefore, exploring the genetic basis of wheat Gh is of great significance for improving wheat quality and commodity properties of wheat.

The most famous loci determining Gh is the *Hardness* (*Ha*) on chromosome 5DS, including *Pina-D1*, *Pinb-D1*, and *Gsp-1* genes (Bhave and Morris, 2008). These three genes encode PINA, PINB, and GSP-1, respectively, which are responsible for the texture of the endosperm and form a friabilin fraction present on the surface of water-washed starch granules (Gupta et al., 2008; Pasha et al., 2010; Martin et al., 2017). The homoeologous loci on chromosomes 5A and 5B lack both *Puroindoline* genes, which having been deleted during durum evolution. In contrast, *Gsp-1* is retained in durum wheat and all three sub-genomes in common wheat (Turnbull et al., 2003; Chantret et al., 2005). The wild-type genotypes (*Pina-D1a* and *Pinb-D1a*) commonly represented the soft-textured grains, whereas mutations in either *Pina-D1a* or *Pinb-D1a* usually lead to hard-textured grains (Bhave and Morris, 2008). Previous studies have reported thirteen allelic variants at the *Pina-D1* locus (*Pina-D1b*, *f*, *k*–*n*, and *p* -*v*) and sixteen allelic variants at the *Pinb-D1* locus (*Pinb-D1b*-*g*, *l*, *p*-*w*, *aa*, and *ab*), including nucleotide mutations, point mutations, and frameshift mutations in common wheat (Bhave and Morris, 2008; Ikeda et al., 2010; Ramalingam et al., 2012; Chen et al., 2013).

In addition to the *Ha* locus, the novel genetic factors other than *Pin* genes remain to be exploited. Numerous grain hardness-associated quantitative trait loci (QTLs) have been identified on almost all chromosomes of wheat (Martin et al., 2001; Turner et al., 2004; Boehm et al., 2018; Kumar et al., 2019; Tu and Li, 2020). For instance, six QTLs were detected for grain hardness on 1B, 4B, 5B, 2D, 4D, and 5D chromosomes by a genome-wide association study (GWAS), with phenotypic variation explanation of 3.7%-50.31% (Wang et al., 2012). Ibba et al. (2019) used 8537 markers to identify two major significant regions related to Gh which were identified on chromosomes 3AL and 6AS each responsible for an additive effect of ~6 hardness index units. However, few QTLs were stably expressed with high phenotypic (>10%) variation in multiple environments, which mean most of them were moderate or environment-special Gh-related QTL and hindered their possible applications in wheat breeding programs.

Moreover, the traditional QTL mapping methods depending on the genotyping all individuals in biparental mapping population, which is time-consuming, laborious, and costly due to the enormous size and complexity of the wheat genome (~ 17 Gb) of which the exome constitutes less than 5% (International Wheat Genome Sequencing Consortium, 2014). To resolve these issues, targeted sequencing approaches, such as exome capture, were developed and used to facilitate mapping major QTLs. Among them, exome sequencing to major loci location restricts attention only to the genomic fraction that encodes for mRNA and eventually a phenotype (Kaur and Gaikwad, 2017). The technique has advantage of being extraordinarily quick, simple, inexpensive, and requires small amount of input DNA (<1–3 mg) (Mertes et al., 2011). For example, by the exome capture sequencing of bulked segregant analysis (BSE-Seq) method, Mo et al. (2018) quickly identified a clear peak region on chromosome arm 4BS associated with increased plant height. Yu et al. (2022) identified three major stable locus controlling spike length and spike compactness on chromosomes 2A and 2D. Martinez et al. (2020) fine-mapped the ABA-hypersensitive mutant ERA8 in a wheat backcross population to the *TaMKK3-A* locus of chromosome 4A. Therefore, BSE-Seq provides other efficient approach to identify favorable alleles of quantitative traits and accelerate the process of wheat breeding. However, BSE-Seq commonly only target one specific trait, the traditional QTL mapping can efficiently detect genome regions for multiple traits, especially the correlated traits.

In present study, we developed a recombinant inbred line (RIL) population derived from the cross of Zhongkemai138 (ZKM138) and Chuanmai44 (CM44) for both QTL mapping and BSE-Seq. The aims of this study were to (i) identify QTL controlling quality-related traits, especially the Gh related trait, and evaluate effect of major QTL; (ii) develop KASP markers linked to the detected major QTL and analyze its potential breeding value; (iii) predict candidate genes for the detected novel major QTL.

## Materials and methods

### Plant materials and field trials

A population of 170 recombinant inbred line (RIL) lines, generated by the single seed descent method from a cross of Zhongkemai138 (ZKM138) × Chuanmai44 (CM44) (indicated as BC-RIL), was used in this study. ZKM138 and CM44 are both widely adaptable varieties in Sichuan Province, released by Chinese Academy of Sciences Chengdu Institute of Biology (CIBCAS) and Sichuan Academy of Agricultural Sciences, respectively. The parents and BC-RILs were planted and evaluated in seven environments (year × locations × treatments) as follows: 2018-2019 in Shuangliu (SL, 103 ° 52’E 30°34’N) with high nitrogen (HN) (1E); 2018-2019 in Shifang (SF, 104 ° 11’E, 31°6’N) with HN (2E) and LN (3E); 2019-2020 in Shifang with HN (4E) and LN (5E); 2019-2020 in Shuangliu with HN (6E) and LN (7E). In the high nitrogen (HN) plots, the organic fertilizer (300 kg ha^-1^), nitrogen fertilizer (120 kg ha^-1^), phosphate fertilizer (50 kg ha^-1^), and potash fertilizer (50 kg ha^-1^) as a base fertilizer were applied only before sowing. In the low nitrogen (LN) plots, no nitrogen fertilizer was applied during the growing period. Each RIL was single-seed planted in two-row plot with a length of 1 m with 20 seeds per row, at a row spacing of 0.25 m. Additionally, 144 derivatives of ZKM138 also developed by CIBCAS were used to further validate the target QTL and assess its application potential in our actual breeding process. Three hundred and seventy-three worldwide varieties (**Supplementary Table 6**) were employed to evaluate the distribution of the target locus and its potential application value. Field management and disease control were performed according to the common practices for wheat production.

### Phenotypic evaluation and statistical analysis

At maturity, at least six representative plants of each line as the repetitions were harvested randomly to measure the phenotype of grain hardness (Gh) and the other 12 quality-related traits, including grain water absorption (Abs), moisture content (Mc), grain protein content (Gpc), grain starch content (Gsc), grain wet gluten content (Wgc), sedimentation volume (Sv), test weight (Tw), dough development time (Ddt), dough stability time (Dst), tensile elongation (Te), tensile area (Ta), maximum tensile resistance (Mtr). These traits were measured by near-infrared reflectance spectroscopy (NIRS) on a Perten DA-7200 instrument (Perten Instruments, Huddinge, Sweden) and expressed on a 14% moisture basis. The correlation between NIRS and traditional methods was confirmed by previous studies (Li et al., 2012; Asif et al., 2015). Especially, the importance of NIRS on wheat grain hardness has been proved (Sun et al., 2009) as a powerful method for measuring grain hardness and other quality traits in wheat.

Basic statistical analyses, frequency distribution, and correlation coefficients among traits were conducted using SPSS version 25.0 for Windows (IBM SPSS, Armonk, NY, United States). The best linear unbiased estimator (BLUE) was calculated using QTL IciMapping software. The broad-sense heritability (*H^2^*) was estimated according to the method described by previous studies (Smith et al., 1998; Muqaddasi et al., 2019).

### SEM observation of grains

A transverse section of grain was observed by an Apre S scanning electron microscope (ThermoFisher) after the grain was snapped in the middle according to previous report (Moeko et al., 2018). These conditions of SEM observation were similar to those described in previous report (Moeko et al., 2018).

### Genetic map construction and quantitative trait loci detection

A genetic linkage map, using 993 bin markers from the Wheat 50K SNP array, previously constructed for the BC-RIL population was adopted for QTL analysis in this study. This genetic map was spanned 1936.59 cM across the 21 wheat chromosomes with an average interval of 0.47 cM per marker (Liu et al., 2022). JoinMap 4.1 and IciMapping 4.2 (Meng et al., 2015) were used for genetic construction. The function of ‘Population’ in JoinMap 4.1 was used to create groups with a limit of detection (LOD) score values ranging from 2 to 10. The Kosambi mapping function was used to order the bin markers, with the parameters being set as LOD ≥ 7 and round = 3, in JoinMap 4.1. Detection of QTL was based on the Biparental Populations (BIP) module for inclusive composite interval mapping (ICIM), with a walking step = 0.001 cM and PIN = 0.001, and a test with 1,000 permutations was used to identify the LOD threshold. A LOD value ≥ 2.5 was used to detect putative QTLs. Moreover, a QTL with a LOD value ≥ 3.5 and phenotypic variation > 10% (on average) that was detected in more than three environments was considered a major stable QTL (Li et al., 2022). QTLs were named according to the International Rules of Genetic Nomenclature, where “cib” represents Chengdu Institute of Biology (Boden et al., 2023). If the confidence intervals of corresponding QTLs overlapped, these QTLs were considered and named one QTL.

### Development of Kompetitive Allele-Specific PCR markers

According to the candidate regions obtained by BSE-Seq analysis, SNP/InDel between the parents and the extreme pools were converted to Kompetitive Allele Specific PCR (KASP) markers for QTL confirmation and linkage analyses. KASP primers were developed following standard guidelines. The allele-specific primers were designed to carry FAM and HEX tails at the 3’ end of the targeted SNP. Common primers were designed using the online primer design pipeline PrimerServer (http://202.194.139.32/PrimerServer). According to the previous report (Li et al., 2022), the reaction conditions and systems of the polymerase chain reaction (PCR) system and the KASP arrays were conducted.

### BSE-seq analysis

The genomic DNA of 40 extreme phenotype individuals from 14-day-old seedlings of the BC-RILs and parents was extracted by CTAB method, and then the genomic DNA was bulked in an equal ratio to generate the two DNA pools, including 20 individuals (named MAX-7) with high grain hardness and 20 individuals (named MIN-7) with low grain hardness. The selected proportion comprised 11% at each tail for extreme phenotypes, which was presumed to provide a 95% probability of detecting QTLs with large effects (Sun et al., 2009). Exome capture sequencing and analysis of four DNA libraries (ZKM138, CM44, MAX-7, and MIN-7) were carried out by OE Biotech Company (Shanghai, China).

Raw sequence reads were filtered using Fastp (v0.12.4) to remove the low-quality reads and adapters used. The high-quality reads were then aligned to IWGSC RefSeq v1.0 genome. After that, raw cohort vcf was worked out with GATK (v4.0.10.1) (Mccormick et al., 2015). The minimum-mapping quality parameter was set as 30 for only high-quality alignment reads used to call variants. SNP calling and density analysis were carried out using sliding window calculation based on the reference of Takagi et al. (2013). The data filtering parameters were set as AF (Allele Frequence) <0.3 or >0.7. The data of BSE-Seq was deposited in the NCBI repository, accession number: PRJNA899434.

### Prediction of candidate gene

The physical positions of major QTL were obtained by aligning the sequences of flanking markers with the wheat reference genome assembly constructed in the cv. Chinese Spring sequence (IWGSC RefSeq v1.0) with a BLAST search. Afterward, genes within the physical regions were extracted using Interval Tools of the Wheat Omics and their annotated functional descriptions were retrieved from UniPort (https://www.uniprot.org/). The expression data of each gene in different tissues (roots, leaves/shoots, spike, and grain) was retrieved from Wheat Expression Browser (http://www.wheat-expression.com/). And the expression pattern analysis of these genes was performed and presented in the HeatMap drawn by TBtools software (Chen et al., 2020). Moreover, based on the whole genome re-sequencing data of ZKM138 and CM44, SNPs and Indels of these genes in the target region were obtained and analyzed.

## Results

### Phenotypic evaluation and correlation analysis

**Figure 1:**
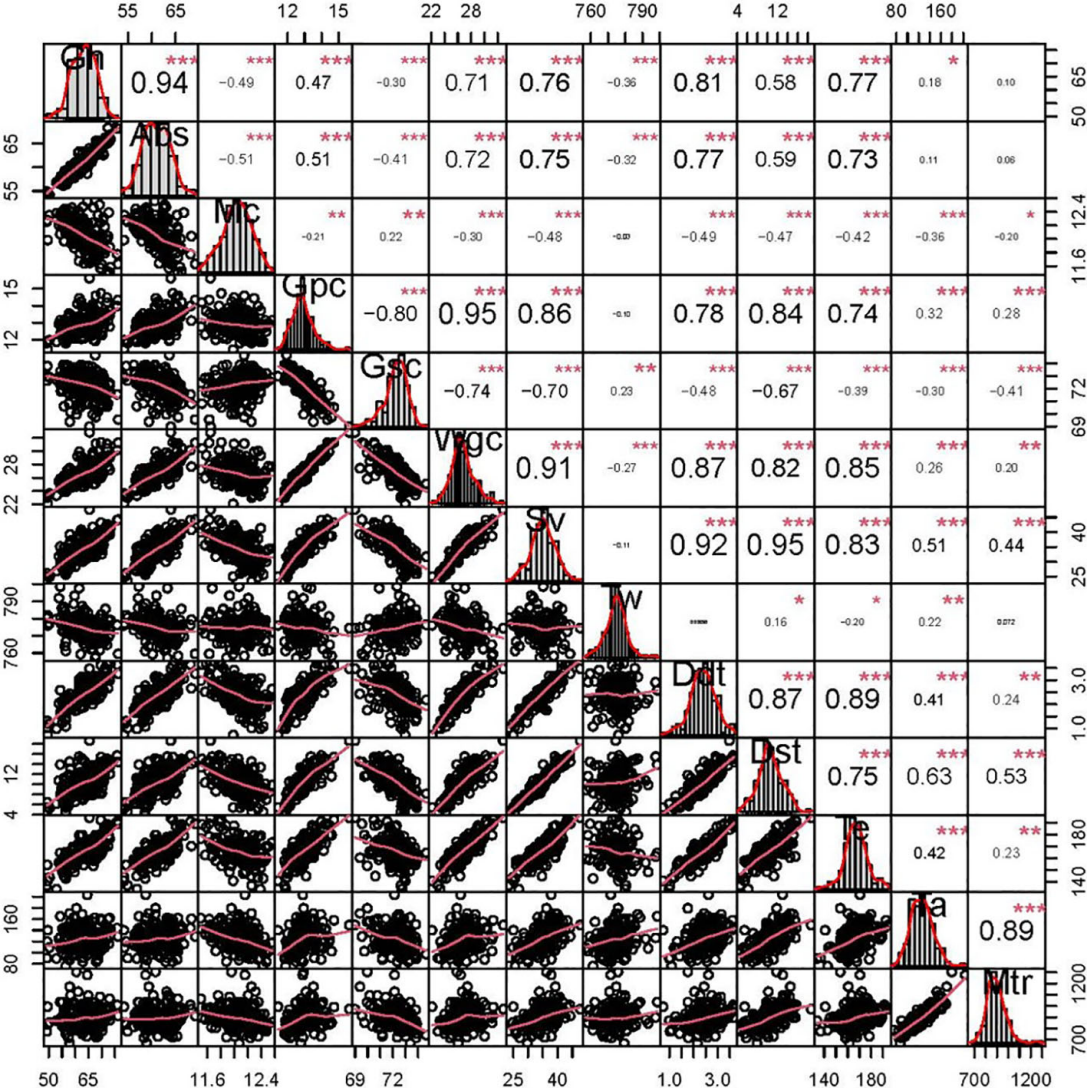
Phenotypic distributions and correlation coefficients of quality-related traits in the BC-RIL population based on BLUE data. *, **, and *** represent significance at *P* < 0.05, *P* < 0.01, and *P* < 0.001, respectively. grain hardness (Gh), grain water absorption (Abs, %), moisture content (Mc, %), grain protein content (Gpc, %), grain starch content (Gsc, %), grain wet gluten content (Wgc, %), sedimentation volume (Sv, mL), test weight (Tw, g L^-1^), dough development time (Ddt, min), dough stability time (Dst, min), tensile elongation (Te), tensile area (Ta), and maximum tensile resistance (Mtr).

ZKM138 exhibited significant higher Gh and Abs than CM44 in all environments (**Supplementary Figure 1**; **Supplementary Table 1**). These measured traits exhibited a normal distribution in the BC-RIL population, and the frequency distribution showed continuous variation, indicating that these traits were determined by multiple genes (**Figure 1**; **Supplementary Table 1**). Broad-sense heritability ranged from 0.53 (Gsc) to 0.83 (Gh and Abs), indicating most of them, especially Gh and Abs, were mainly controlled by genetic factors (**Supplementary Table 1**).

Phenotypic correlations between the all detected quality-related traits are showed in **Figure 1**. Gh was significantly positively correlated with Abs (*r* = 0.94), and the significantly negative correlation coefficients were observed for Gh-Tw (*r* = -0.36) and Gh-Gsc (*r* = -0.30), respectively. In addition, Gpc was significantly negatively correlated with Gsc (*r* = -0.80), consistent with their well-known trade-off (Fan et al., 2022).

### QTL detection

A total of 153 QTLs for these thirteen traits were detected in BC-RIL population and located on all the 21 chromosomes accounting for 1.16%-18.2% of the phenotypic variation (**Supplementary Table 2**). Among them, 24 QTLs were classified as the major QTLs (PVE > 10%), of which 11 major stable QTLs were detected in multiple environments and described in detail.

Two major QTL for Ta and Mtr (*QTa.cib-4A* and *QMtr.cib-4A*) were located on chromosome 4A and stably detected in three environments, explaining 12.12% and 10.72% of the phenotypic variance, respectively. On chromosome 4D, *QDdt.cib-4D* for Ddt was detected in three environments and the BLUE dataset with the LOD values ranging from 3.93 to 6.47, explaining 7.80%-10.60% of the phenotypic variance. One major QTL for Gh (*QGh.cib-5D.1*) was discovered on chromosome 5D in six environments, accounting for 12.22% of the phenotypic variation, and collocated with two major stable QTLs for Mc and Abs (*QMc.cib-5D* and *QAbs.cib-5D*). The major QTL *QDst.cib-6D2* for Dst was detected in three environments and explained 7.62%-12.62% of phenotypic variance. On chromosome 7B, two major stable QTLs for Wgc and Ddt (*QWgc.cib-7B* and *QDdt.cib-7B*) expressing in four and three environments, respectively, were collocated with the LOD values of 5.02 and 5.48, explaining 10.39% and 10.13% of the phenotypic variance, respectively, with the positive alleles of both loci contributed by CM44.

On chromosome 7D, two major QTLs were detected in all environments and the BLUE dataset, affecting Gh (*QGh.cib-7D*) and Abs (*QAbs.cib-7D*), and explained 13.74% and 13.2% of the phenotypic variance. At this location, a ZKM138-derived alleles enhanced both Gh and Abs (**Supplementary Table 2**).

### QTL clusters

**Figure 2:**
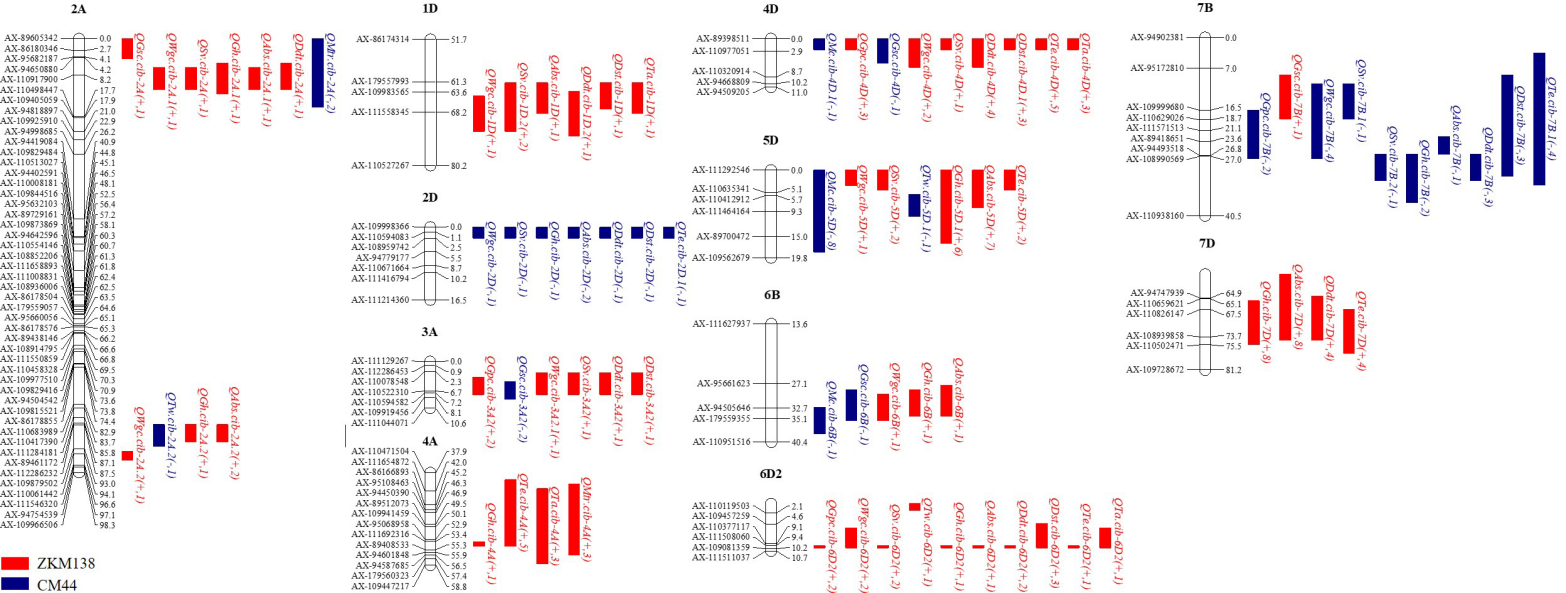
Summary of QTL clustered detected in this study. The brackets after the QTL name follow additive effect and number of environments. The QTL in red and blue represented the positive allele of QTL derived from ZKM138 and CM44, respectively. The QTLs in bold are major QTLs.

In this study, 12 intervals clustering (C1-C12) three or more additive QTLs were observed (**Figure 2**; **Supplementary Table 3**). They were mainly mapped on 11 chromosomes. Seven of them contained at least one major stable QTL that could be detected repeatedly in more than three environments. C3 located at 703-755 Mb on chromosome 2A and contained a major QTL for Gh (*QGh.cib-2A.2*). C6 contained two major stable QTLs for dough rheological properties (*QTa.cib-4A* and *QMtr.cib-4A*) and two moderate QTL for Gh and Te (*QGh.cib-4A* and *QTe.cib-4A*), with positive allele derived from ZKM138. C7, involving the famous semi-dwarfing gene *Rht-D1*, was clustered by nine QTLs affecting quality traits, including *QMc.cib-4D.1*, *QGpc.cib-4D*, *QGsc.cib-4D*, *QWgc.cib-4D*, *QSv.cib-4D*, *QDdt.cib-4D*, *QDst.cib-4D.1*, *QTe.cib-4D*, and *QTa.cib-4D*. C8 had three major stable QTLs for Gh (*QGh.cib-5D.1*), Abs (*QAbs.cib-5D*), and Mc (*QMc.cib-5D*), and probably associated with the famous hardness *Pin* genes. C10 contained one major stable QTLs for Dst (*QDst.cib-6D2*) and eight QTLs (*QGpc.cib-6D2*, *QWgc.cib-6D2*, *QSv.cib-6D2*, *QGh.cib-6D2*, *QAbs.cib-6D2*, *QDdt.cib-6D2*, *QTe.cib-6D2*, *QTa.cib-6D2*), with the ZKM138-derived allele increasing all these traits. Furthermore, C12 had two major stable QTLs for Gh and Abs (*QGh.cib-7D* and *QAbs.cib-7D*) with high phenotypic variation, implying a strong relation to wheat processing.

### Additive effects of *QGh.cib-5D.1* and *QGh.cib-7D* on grain hardness

**Figure 3:**
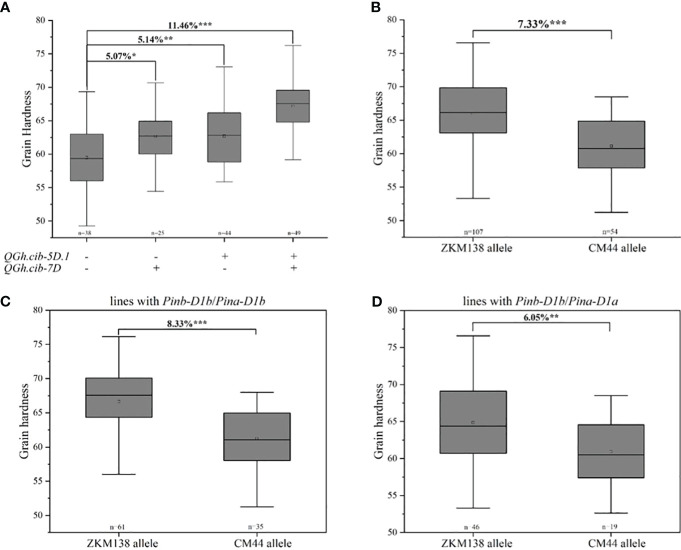
Additive effects of major QTL on grain hardness. **(A)** Pyramiding effect analysis of *QGh.cib-5D.1* and *QGh.cib-7D* on grain hardness. Effects of *QGh.cib-7D* on grain hardness based on BC-RILs **(B)**, the lines with *Pinb-D1b*/*Pina-D1b* **(C)**, and *Pinb-D1b*/*Pina-D1a* **(D)**, respectively. Symbols + and – represent lines with and without the positive alleles (ZKM138 allele) for the target QTL based on the flanking marker of the corresponding QTL, respectively. ‘n’ denotes the number of lines in each panels. *, **, and *** represent significance at *P* < 0.05, *P* < 0.01, and *P* < 0.001, respectively.

In this study, two major QTLs for grain hardness stably expressed on chromosomes 5D and 7D in all environments, which were two major stable QTLs for Gh in this study. ZKM138-derived allele contributed the increasing Gh at both loci, which might provide the major genetic basis to ZKM138 for its higher Gh. The pyramiding effects of two QTLs on grain hardness were analyzed (**Figure 3A**). The lines with a positive allele, ie., ZKM138-derived allele, at either of *QHa.cib-5D.1* or *QGh.cib-7D* significantly increased grain hardness by 5.07%-5.14% relative to lines without any positive alleles; and the lines with ZKM138-derived allele at both loci significantly increased grain hardness by 11.46%. These results indicated that these two loci had a pyramiding effect on grain hardness, which could be applied together for grain hardness selection and breeding.

Considering the physical location of *QHa.cib-5D.1* was located on chromosome 5DS and was overlapped with *Pin* genes. According to functional markers genotyping reported in previous studies (Giroux and Morris, 1997; Li et al., 2006), the results showed that both ZKM138 and CM44 carried *Pinb-D1b*, while ZKM138 carried *Pina-D1b* and CM44 carried *Pina-D1a*, and proved that it was the major contributor for increasing Gh at *QHa.cib-5D.1* (**Supplementary Figure 2**).

Only for *QGh.cib-7D*, the lines with ZKM138-derived allele had significantly higher Gh (*P* < 0.001) than the lines carrying CM44-derived allele (**Figure 3B**). To further elucidate the separate and effective effect of *QGh.cib-7D* other than *Pina* on grain hardness, the population were divided into 2 groups, according to the lines whether carrying *Pina-D1a* (**Figures 3C, D**). The results showed that the lines harbored ZKM138-derived allele at *QGh.cib-7D* increasing grain hardness 8.33% when *Pinb* and *Pina* loci were *Pinb-D1b* and *Pina-D1b*, respectively, i.e., under the background with *Pinb-D1b*/*Pina-D1b* (**Figure 3C**); and under the background with *Pinb-D1b*/*Pina-D1a*, the lines harbored ZKM138-derived allele at *QGh.cib-7D* also increased grain hardness 6.05% (**Figure 3D**). In summary, *QGh.cib-7D* had a strong association with grain hardness, independent of the famous *Pina*.

### BSE-seq analysis for grain hardness

Exome capture and high-throughput sequencing of the four bulked pools, MAX-7, MIN-7, and parents ZKM138 and CM44 were performed to identify the genomic regions associated with Gh, and the results were compared with the Chinese Spring (CS) reference genome v1.0 by IWGSC. The total number of clean reads after filtering was 714061024 and the number of clean bases obtained from a total of six pools was 106.98 Gb, with clean reads ranging from 176218966 to 202584102 for a single pool, which indicated that the sequencing data were available for the subsequent analysis. The coverage of the parents in the wheat genome was more than 90%, and the average depth was more than 25×. Furthermore, the average depth of the two pools of data (MAX-7, MIN-7) was 50.99× and 58.50×, with the coverage in the Chinese Spring genome of 96.06% and 96.73%, respectively (**Supplementary Table 4**). These results indicated that the BSE-Seq assays among the pools were efficient in the present study.

The SNP/InDel index algorithm was used to determine the candidate region for grain hardness. With the SNP-index algorithm, a total of 1056 high-quality SNPs were identified between two pools, of which 435 (41.19%) SNPs were concentrated in the 46.05-60.55 Mb of chromosome 7D (**Supplementary Figure 3**), consistent with the traditional QTL mapping results (**Supplementary Table 2**).

### Effects of *QGh.cib-7D* on grain hardness and other quality traits

**Figure 4:**
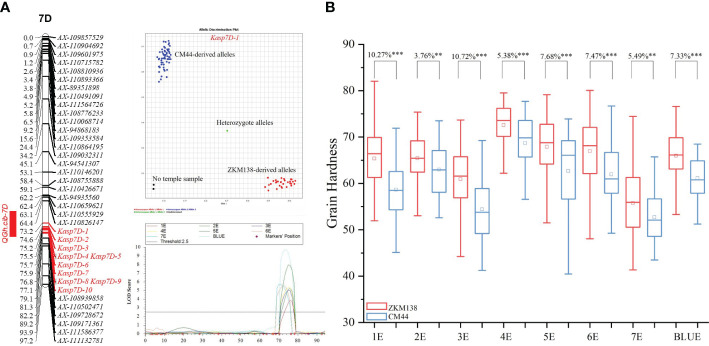
Genetic maps of *QGh.cib-7D* **(A)** and its effects on grain hardness **(B)** in BC-RIL populations. The developed 10 KASP markers for *QGh.cib-7D* were integrated onto the genetic map. ZKM138 and CM44 indicate the lines with the alleles from ZKM138 and CM44, respectively. *, ** and *** represent significance at *P* < 0.05, *P* < 0.01, and *P* < 0.001, respectively.

According to polymorphic SNPs in the target region of BSE-Seq, 48 KASP markers were developed and genotyped in BC-RILs. Finally, a new integrated genetic map for the candidate region of *QGh.cib-7D* was reconstructed with ten KASP markers (**Supplementary Table 5**). After remapping based on the new map, the original candidate region was narrowed from 10.91 Mb (54.59-65.50) to 2.71 Mb (58.64-61.35 Mb) (**Figure 4A**).

According to the narrowed map, the lines carrying ZKM138 allele had higher Gh than that of CM44 allele, with differences of 3.76%-10.72% (*P* < 0.01) under all the eight detected environments (**Figure 4**), which was consistent with the SNP genotyping results, using *Kasp7D-1* as the diagnostic marker. In addition, the effects of *QGh.cib-7D* on other quality traits were analyzed in the BC-RIL population. The results revealed that *QGh.cib-7D* significantly increased grain water absorption (3.87%) while it did not influence the grain starch content and protein content (**Supplementary Figure 4**).

### Distribution and transmissibility of the positive allele of *QGh.cib-7D*

**Figure 5:**
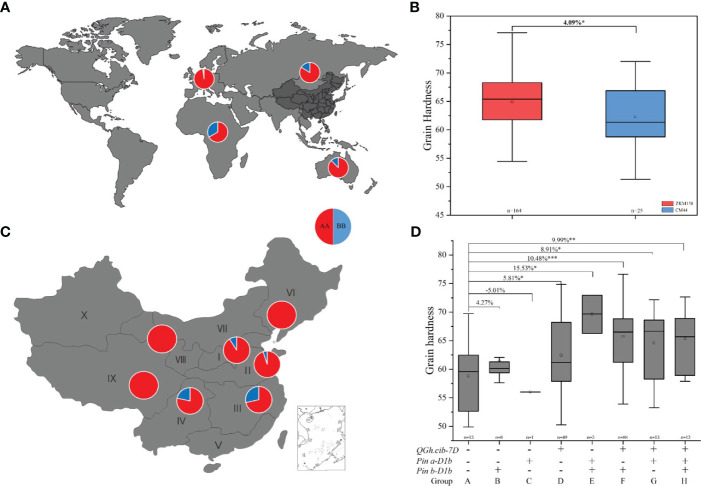
Geographic patterns of the genotype of *QGh.cib-7D* in worldwide varieties **(A)** and Chinese varieties **(B)**. **(C, D)** Additive effects of *QGh.cib-7D*, *Pina*, and *Pinb* on grain hardness in Chinese varieties. AA and BB represent positive allele of *QGh.cib-7D* derived from ZKM138 and CM44, respectively. Symbols + and – represent lines with and without the positive alleles for the target QTL based on the flanking marker of the corresponding QTL, respectively. ‘n’ denotes the number of varieties in each panels. *, **, and *** represent significance at *P* < 0.05, *P* < 0.01, and *P* < 0.001, respectively.

To further explore the potential application value of *QGh.cib-7D* on wheat grain hardness selecting and improving, the genotypes of 373 common wheat varieties from different countries were analyzed. As shown in **Figure 5A**, the ZKM138-derived allele existed with high-frequency presenting in 320 of 373 varieties (85.79%), spreading in four continents, including Asia, Europe, Oceania, and Africa (**Figure 5A**; **Supplementary Table 6**). In China, 164 (86.77%) varieties of these 189 Chinese varieties, containing this ZKM138-derived allele, which significantly increased Gh by 4.09% (**Figure 5B**). And only 25 varieties contained CM44-derived allele, of which 18 varieties were from Southwestern Autumn-Sown Spring Wheat Zone, a region in southern of China with famous distillery (**Figure 5C**).

Moreover, besides *QGh.cib-7D* locus, considering that the effect of *Pina* and *Pinb*, there were eight, one, and forty-nine varieties only containing *Pinb-D1b* (group B), *Pina-D1b* (group C), or ZKM138-derived allele (group D), respectively (**Figure 5D**). And there were three, eighty-eight, and thirteen varieties harboring *Pina-D1b*/*Pinb-D1b* (group E), ZKM138-derived allele/*Pinb-D1b* (group F), and ZKM138-derived allele/*Pina-D1b* (group G), that significantly increased grain hardness by 8.91%-15.53% (**Figure 5D**). Lastly, thirteen varieties contained all the three alleles (group H) and significantly increased grain hardness by 9.99%. It is noteworthy that based on the background of positive allele of *QGh.cib-7D*, the varieties with *Pina-D1b* (group G) had higher grain hardness compared to the varieties with *Pinb-D1b* (group F) (**Figure 5D**).

### Potential candidate genes analysis of *QGh.cib-7D*

**Figure 6:**
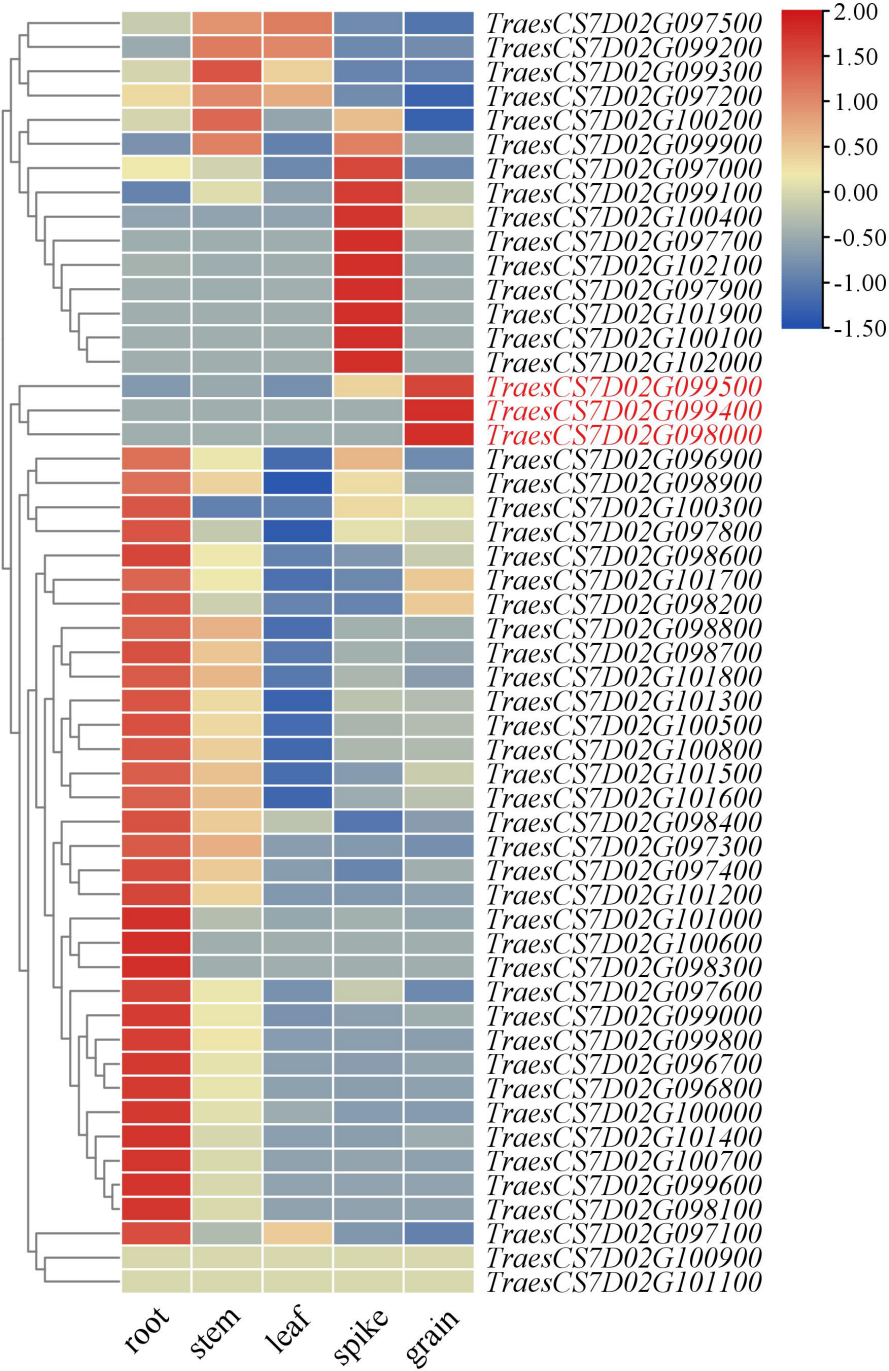
Expression patterns of candidate genes in the physical interval of *QGh.cib-7D* in different tissues. Genes in red represents genes specifically expressed in the spike and grain; the data of expression profiles were obtained from the public online database.

The candidate genes in *QGh.cib-7D* were finally mapped to a 2.71 Mb physical interval, which contained 53 high-confidence genes (**Supplementary Table 7**). Analysis of the spatiotemporal expression patterns showed that three genes (*TraesCS7D02G099400*, *TraesCS7D02G098000*, and *TraesCS7D02G099500*) that were highly and specifically expressed in grain, which might be probably involved in grain development (**Figure 6**). *TraesCS7D02G099400* encoded 30S ribosomal protein S3, and *TraesCS7D02G098000* encoded basic blue protein while *TraesCS7D02G099500* encoded putative oligopeptide transporter. Based on re-sequencing results of parents, 13 synonymous SNPs and 5 nonsynonymous SNPs were detected in the coding region of *TraesCS7D02G099500*.

## Discussion

### *QGh.cib-7D* might be a novel locus for grain hardness

Grain hardness is one of the most important characteristics for milling and baking quality of wheat (Pasha et al., 2010). In the present study, almost all quality-related traits, except Mc and Gsc, were significantly positively correlated with each other, with the highest correlation coefficient between Gh and Abs (*r* = 0.94) (**Figure 1**), consistent with previous studies (Cui et al., 2016; Fan et al., 2022). Gh is not only controlled by the main effect genes (*Ha*) on chromosome 5DS but also through minor genes on other chromosomes (Tu and Li, 2020), as well as the potential unexcavated. In this study, twelve clusters for quality-related traits were detected, but most of them were previously reported, except C12 harboring *QGh.cib-7D* for Gh. For example, C1, located in the interval of 363-415 Mb on chromosome 1D and harbored six QTLs for Dst, Abs, Ta, Sv, Ddt, and Wgc, which was consistent with reported QTLs for quality-related traits (Guo et al., 2020). Comparative analysis indicated that it overlapped with *Glu-1D* consisting of x-type and y-type, which form different cross-chains and structures in the gluten, thus regulating the elasticity, ductility, and viscosity of the dough (Kong et al., 2004). C3 contained a major QTL (*QGh.cib-2A.2*) and 3 moderate QTLs (*QAbs.cib-2A.2*, *QTw.cib-2A.2*, and *QWgc.cib-2A.2*), was physically overlapped with *Ppo-A1*, a core gene regulating polyphenol oxidase and thus influencing end-use quality (Beecher and Skinner, 2011). *Waxy* (Chang et al., 2021) and *BGC1* (Chia et al., 2020), associated with starch synthesis, was located in the region of C6, which harbored QTLs for *QGh.cib-4A*, *QTe.cib-4A*, *QTa.cib-4A*, and *QMtr.cib-4A*, and thus might be the gene responsible for this QTL cluster. In the C7 cluster associated with *Rht-D1*, multiple QTLs for quality-related traits, such as *QDdt.cib-4D*, *QMc.cib-4D.1*, and *QGpc.cib-4D*, were co-located in this region, indicating that *Rht-D1* may have pleiotropic effects on plant height, yield, and quality (Pearce et al., 2011). These results indicated that the cluster C3 could be used to simultaneously improve several quality-related traits in molecular module breeding. Moreover, C8, located at 1-6 Mb on chromosome 5D, simultaneously harbored three major stable QTL (*QGh.cib-5D.1*, *QAbs.cib-5D*, and *QMc.cib-5D*). As *Pin* genes were reported to regulate grain hardness (Bhave and Morris, 2008), we integrated the functional marker of *Pina* into our genetic map. The result showed that this cluster was tightly linked to *Pina* (**Supplementary Figure 2**) as expected. Consequently, the effect of C8 might be attributed by *Pina*. However, *QGh.cib-7D* and *QAbs.cib-7D* in C12 were detected in all environments with high phenotypic variation and co-located with two stable QTLs (*QDdt.cib-7D* and *QTe.cib-7D*). Comparative analysis revealed that no previously reported QTL or genes for grain hardness were overlapped with or near to this region. Therefore, this interval might be a novel locus for grain hardness.

### The potential breeding value of *QGh.cib-7D* in wheat quality improvement

Manipulation of the level of grain hardness not only modifies milling properties in common and durum wheat lines (Li et al., 2014; Wang et al., 2019a), but also changes some parameters of the end-use quality and storage protein interaction (Kiszonas and Morris, 2018; Wang et al., 2019b). The previous studies reported that *Pin* genes could only explain over 60% of the trait’s variation and hinder the identification of other QTLs with minor effects in the bi-parental mapping population (Sourdille et al., 1996; Campbell et al., 1999). Identifying the key genomic region for Gh and pyramiding the elite alleles through marker-assisted selection could guide our breeding efficiency. In the present study, two major QTLs, *QGh.cib-5D.1* (the *Ha* locus) *QGh.cib-7D*, and 15 QTL regions for grain hardness were identified. This novel QTL, *QGh.cib-7D*, could be simultaneously detected by QTL mapping and BSE-seq, indicating its robust and reliable effect on Gh. Pyramiding analysis revealed that the combination of two major QTLs significantly increased grain hardness (**Figure 3A**). To eliminate the major genetic effects of the *Ha* locus and to reveal the individual effect of *QGh.cib-7D* on Gh, association analysis of *QGh.cib-7D* with Gh revealed that the lines carried positive allele of *QGh.cib-7D* (ZKM138-derived allele) significantly increased grain hardness 6.05%-8.33% based on the BC-RIL background (**Figures 3C, D**), indicating that *QGh.cib-7D* might be useful for Gh selection improvement and breeding.

Converting the significant SNPs into KASP markers is beneficial for marker assisted breeding (Liu et al., 2022). In this study, ten KASP markers were developed and integrated in a new genetic map based on the results of BSE-Seq (**Figure 4A**). Finally, the genomic region of *QGh.cib-7D* was narrowed to a 58.64-61.35 Mb (2.71 Mb) region, and *Kasp7D-1* was regarded as a diagnostic marker, which was used to investigate the distribution (Liu et al., 2022) and transmissibility (Fan et al., 2018). *Pina* and *Pinb* were two most famous and used widely loci for wheat grain hardness selection and breeding (Pasha et al., 2010; Li et al., 2019). As shown in **Figure 5** and **Supplementary Table 6**, if the effect of *QGh.cib-7D* were not taken into account, we could found that 66 of 200 (33%) varieties contained *Pina-D1a* as well as *Pinb-D1a* (*Pina-D1a*/*Pinb-D1a*), which was almost consistent with the reported frequency harboring *Pina-D1a*/*Pinb-D1a* (Li et al., 2019). For example, Li et al. (2019) reported that 39.3% of 107 Chinese accessions harbored both *Pina-D1a* and *Pinb-D1a*. Theoretically, this loci combination (*Pina-D1a*/*Pinb-D1a*) could possibly confer wheat soft grain performance. We indeed noticed that its average Gh value was lower than most groups whose lines harbored only one soft grain genotype at *Pina* or *Pinb*, such as group (B+F) and (C+G). However, there still were numerous materials performing relatively high Gh values in group D, where the increasing-Gh allele existed at *QGh.cib-7D* locus. In other words, the potential unexcavated novel locus also contributes greatly to Gh performance besides *Pina* and *Pinb*. If we did not detect and notice the effect of *QGh.cib-7D* on Gh, it is possible to decrease the efficiency and accuracy of the soft/hard material selection in the MAS process. However, when we calculated the effect of this novel locus for Gh (*QGh.cib-7D*), only 13 of 200 (6.50%) varieties harbored soft Gh-type genotypes at all three loci (*Pina*, *Pinb*, and *QGh.cib-7D* locus), and this group (group A) indeed exhibited the lowest grain hardness, further proving *QGh.cib-7D* might harbor the new gene for Gh with important breeding value. In addition, we found that 11 of them were from the southwest zone in China (**Figure 5B**), which may be related to local dietary and processing preferences and thus presented a relatively higher frequency of soft wheat than other areas. For example, Baijiu, Chinese liquor, has a high reputation and constitutes as an important part of the Chinese dietary profile. The most well-known Baijiu, such as Moutai-jiu, wuliangye-jiu, and luzhoulaojiao-jiu, etc., all produced in this zone. The most raw for Baijiu Jiuqu (starter) were soft wheat (Zheng and Han, 2016). These results also indicated that, with the detection of this new locus on 7D (*QGh.cib-7D*), much less soft wheat varieties with all noticeable soft-Gh genotypes (only 13 materials, 6.5%) actually exist, rather than 33% of 200 materials as we originally thought. It is urgent to cultivate soft wheat to cater to market demand and ensure the diversity of wheat quality. We, therefore, suggested that all these three loci should be simultaneously considered and detected to improve the breeding efficiency in the future soft wheat breeding process.

The texture and smoothness of noodles are connected to grain hardness, and a grain hardness index (HI) value of 60-70 is needed to ensure high-quality noodles and steamed bread (Wang et al., 2022). In this study, also 13 of 200 (6.50%) varieties (group H) harbored the positive of *QGh.cib-7D* and *Pina-D1b*/*Pinb-D1b*, which indeed show higher grain hardness. Interestingly, the combination of any two loci of these three loci (Group E, F and G) still could significantly improve grain hardness for efficient selection of hard wheat varieties, which could save effort to pyramid all the three hard-wheat alleles (*QGh.cib-7D*, *Pina-D1b* and *Pinb-D1b*) when hard wheat MAS breeding.

The negative yield-quality correlation has long been a key obstacle in wheat breeding programs designed to simultaneously improve yield and quality. Consequently, QTLs involved in the control of kernel quality that are also independent of negative effects on grain yield should be identified to improve this characteristic. The major stable QTL *QGh.cib-7D* was significantly associated with grain hardness and had a significant effect on grain water absorption, but no effect on the major nutritional traits, such as protein and starch content, indicating its potential contribution to processing and end-use quality. On the other hand, notably, 143 of the 144 (99.31%) derivatives inherited the key genomic segment of *QGh.cib-7D* from ZKM138 when we used this variety as the core parent in our small-scale breeding process, in which we commonly focus on the yield performance. As shown in **Supplementary Figure 5**, though lines with positive allele of *QGh.cib-7D* increased grain width and thousand-grain weight, but reduced grain per spike. Finally, they showed no significant difference in grain yield between the lines with two alleles, which implied that this locus had no significant negative effect on final yield performance, which explained the reason for the passive selection of *QGh.cib-7D* and further proving the valuable breeding application potential for directional improving and selecting grain hardness without the effect on yield performance.

### Potential candidate genes for *QGh.cib-7D*

Fifty-three high-confidence genes were identified in the *QGh.cib-7D* interval in the CS genome (**Supplementary Table 6**). Based on expression patterns, gene annotation, ortholog analysis, and haplotype analysis, *TraesCS7D02G099400*, *TraesCS7D02G098000*, and *TraesCS7D02G099500*, associated with grain development, were predicted as candidate genes. *TraesCS7D02G099400* encodes 30S ribosomal protein S3, which may affect the grain hardness to some extent by influencing protein synthesis and cell wall formation (Bommer et al., 1991; Schäfer et al., 2006). *TraesCS7D02G098000* encodes basic blue protein, a relatively small group of proteins known as gliadins that interact with the macromolecular gluten subunit in wheat to promote the formation of viscosity and elasticity in dough (Metakovsky et al., 1997). Gliadins are responsible for giving wheat flour its viscoelastic properties, which make it suitable for baking (Wrigley, 1996). *TraesCS7D02G099500* encodes the oligopeptide transporter, which regulates the absorption and transport process of oligopeptides and serves as a nitrogen source for wheat growth and development (Kumar et al., 2019). The expression level and activity of oligopeptide transporters may affect the nitrogen supply and protein synthesis capacity of wheat, thus affecting wheat quality (Stacey et al., 2006; Liu et al., 2012). Moreover, the re-sequencing data between ZKM138 and CM44 revealed that 13 synonymous SNPs and 5 nonsynonymous SNPs existed in the exons. Thus, these three genes could be considered as a focus for further work on fine mapping and gene cloning.

## Data availability statement

The datasets presented in this study can be found in online repositories. The names of the repository/repositories and accession number(s) can be found in the article/**Supplementary Material**.

## Author contributions

XL: Conceptualization, Data curation, Investigation, Methodology, Software, Validation, Writing – original draft. ZX: Methodology, Resources, Supervision, Writing – review & editing. BF: Data curation, Investigation, Writing – review & editing. QZ: Data curation, Supervision, Writing – review & editing. SG: Data curation, Investigation, Methodology, Writing – review & editing. SL: Data curation, Investigation, Writing – review & editing. YO: Data curation, Investigation, Writing – review & editing. TW: Funding acquisition, Project administration, Resources, Writing – review & editing. XF: Funding acquisition, Project administration, Supervision, Writing – review & editing.

## Supplementary material

Supplementary Figure 1 Scanning electron micrographs of grains in the parents. SEM observation of grain sections, Photos of transverse sections of mature grains are shown for ZKM138 and CM44. LS, large starch granule; BS, broken large starch granule. Bar: 1 cm.

Supplementary Figure 2 Identification of the known gene with *QGh.cib-5D.1*. The brackets after the QTL name follow additive effect and the number of environments.

Supplementary Figure 3 BSE-Seq analysis using the SNP-index algorithm **(A)** and distribution of polymorphic SNPs on each chromosome **(B)**.

Supplementary Figure 4 Effects of *QGh.cib-7D* on quality traits. *, ** and *** represent significance at *P* < 0.05, *P* < 0.01 and *P* < 0.001, respectively.

Supplementary Figure 5 Additive effects of *QGh.cib-7D* on yield-related traits in BC-RIL. *, ** and *** represent significance at *P* < 0.05, *P* < 0.01 and *P* < 0.001, respectively.
