# Supplementary figures and images for: Dissection of a novel major stable QTL on chromosome 7D for grain hardness and its breeding value estimation in bread wheat
Source: Front Plant Sci. 2024 Feb 1;15:1356687. doi: 10.3389/fpls.2024.1356687 (PMC10867189; doi:10.3389/fpls.2024.1356687)

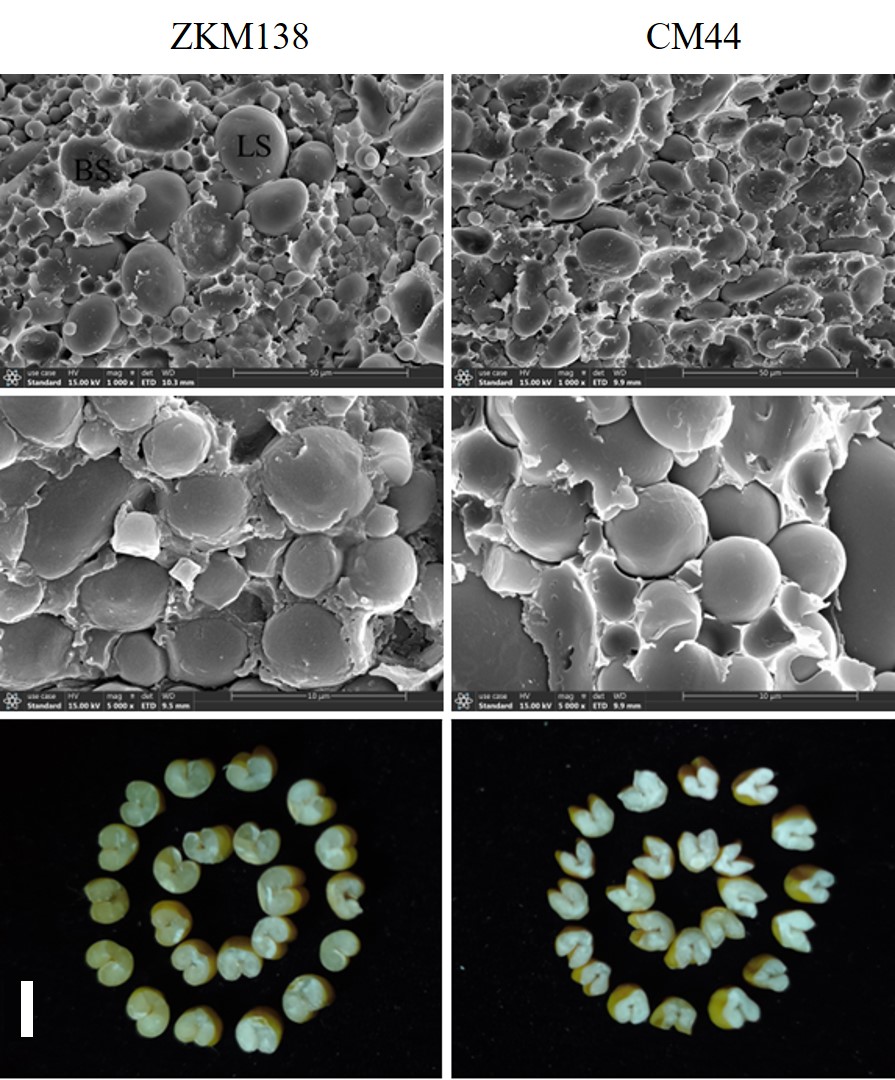

Supplement: Supplementary Figure 1 — Scanning electron micrographs of grains in the parents. SEM observation of grain sections, Photos of transverse sections of mature grains are shown for ZKM138 and CM44. LS, large starch granule; BS, broken large starch granule. Bar: 1 cm. [file Image_1.jpeg]

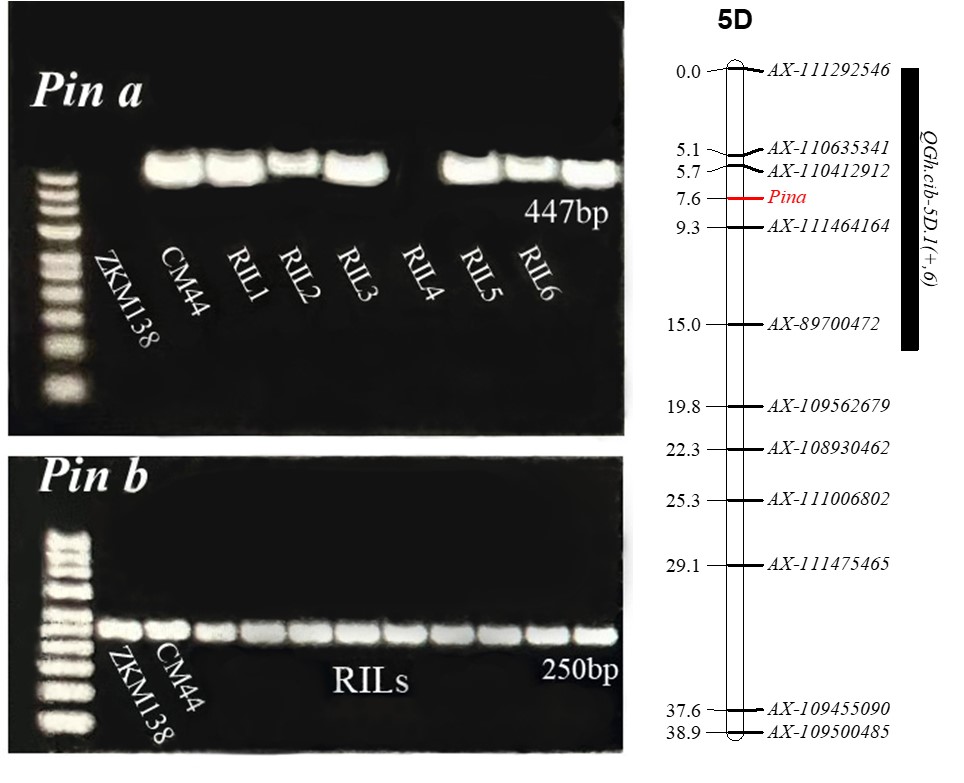

Supplement: Supplementary Figure 2 — Identification of the known gene with QGh.cib-5D.1. The brackets after the QTL name follow additive effect and the number of environments. [file Image_2.jpeg]

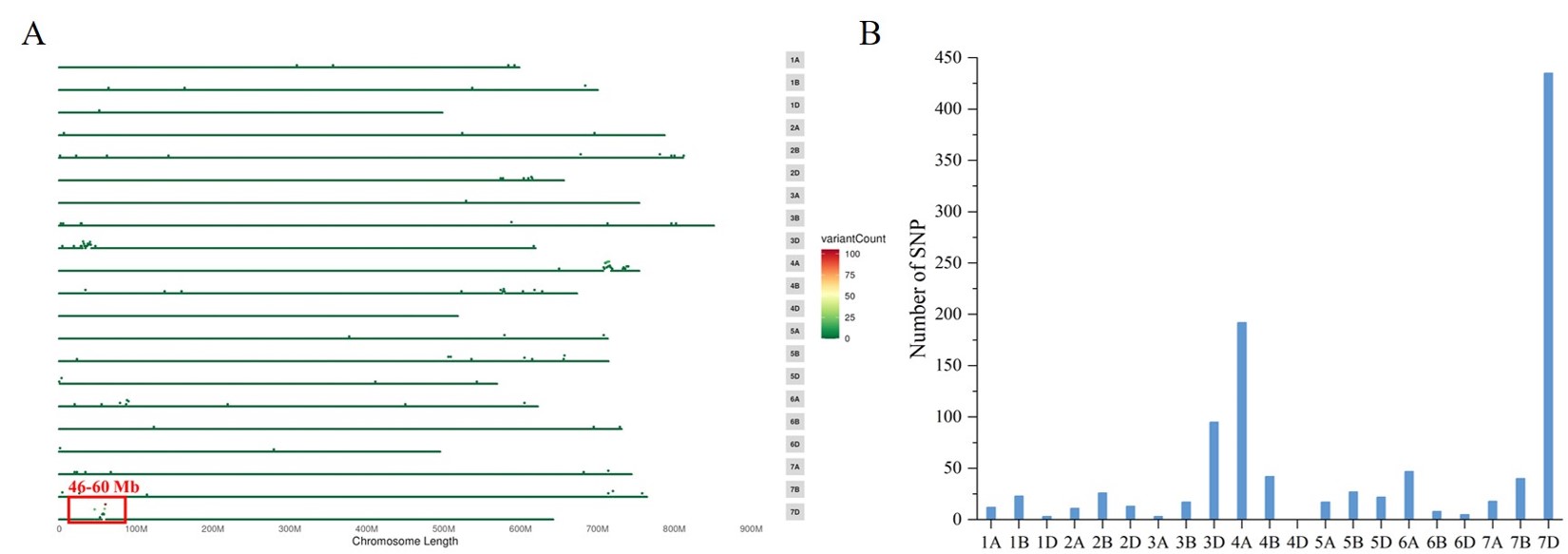

Supplement: Supplementary Figure 3 — BSE-Seq analysis using the SNP-index algorithm (A) and distribution of polymorphic SNPs on each chromosome (B). [file Image_3.jpeg]

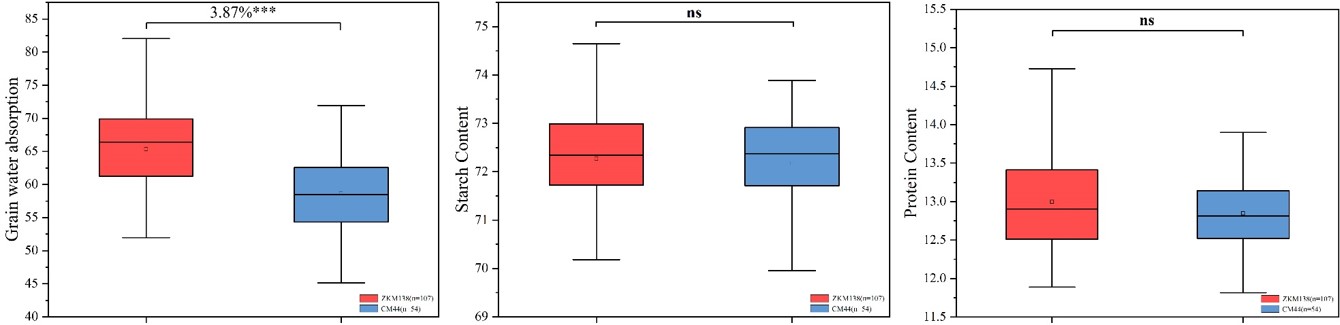

Supplement: Supplementary Figure 4 — Effects of QGh.cib-7D on quality traits. *, ** and *** represent significance at P < 0.05, P < 0.01 and P < 0.001, respectively. [file Image_4.jpeg]

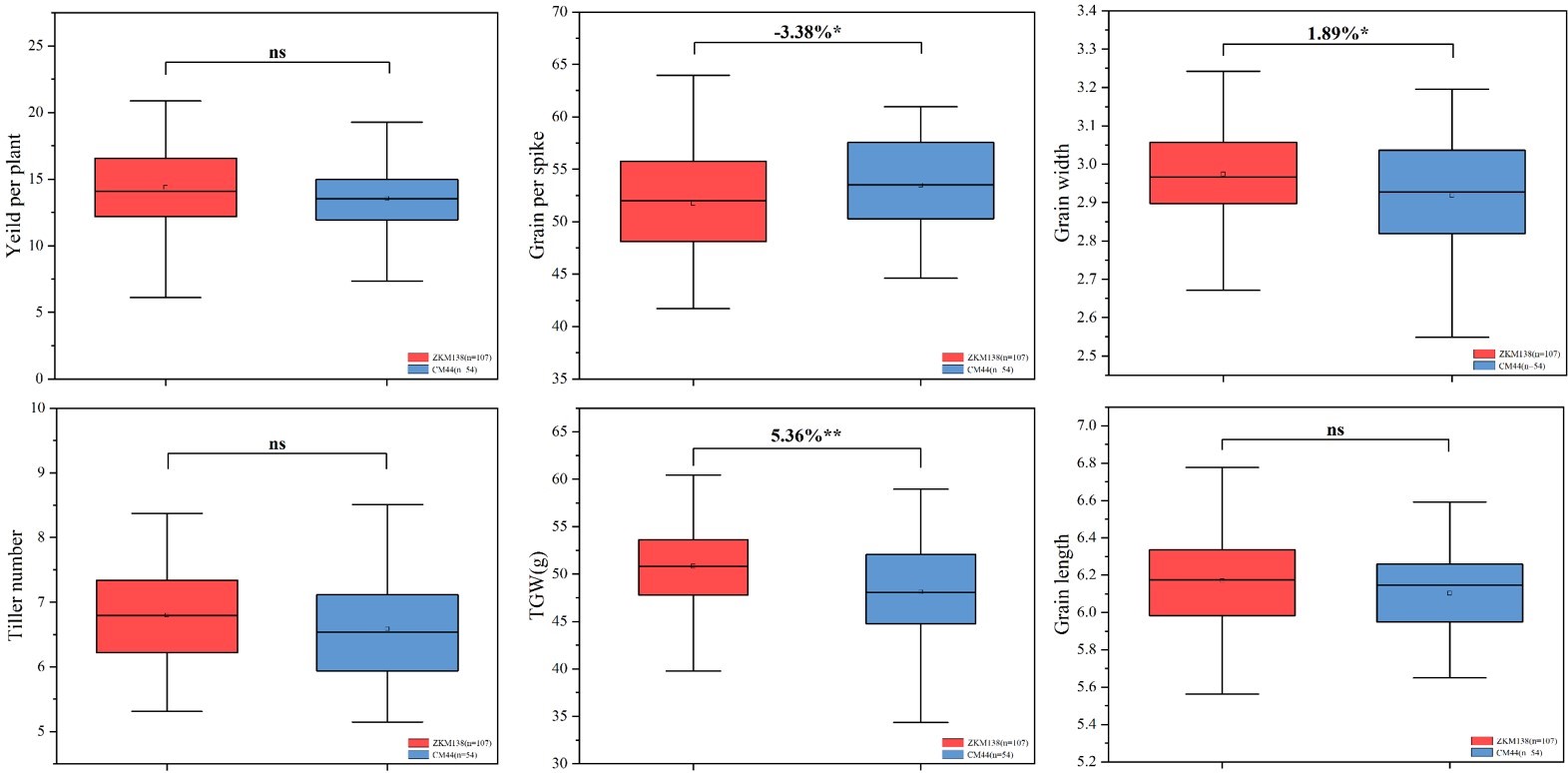

Supplement: Supplementary Figure 5 — Additive effects of QGh.cib-7D on yield-related traits in BC-RIL. *, ** and *** represent significance at P < 0.05, P < 0.01 and P < 0.001, respectively. [file Image_5.jpeg]
